# No Obesity Paradox for Health-Related Quality of Life in Patients with Heart Failure and Reduced Ejection Fraction: Insights from the VIDA Multicenter Study

**DOI:** 10.3390/jcm13247558

**Published:** 2024-12-12

**Authors:** Paula Cassadó-Valls, Cristina Enjuanes, Manuel Anguita, Francesc Formiga, Luis Almenar, María G. Crespo-Leiro, Luis Manzano, Javier Muñiz, José Chaves, Encarna Hidalgo, Raúl Ramos-Polo, Sergi Yun, Núria José-Bazán, Pedro Moliner, Josep Comín-Colet

**Affiliations:** 1 School of Medicine, University of Barcelona, 08097 Barcelona, Spain; paula.cassado@gmail.com; 2 Bio-Heart Cardiovascular Diseases Research Group, Bellvitge Biomedical Research Institute (IDIBELL), L’Hospitalet de Llobregat, 08908 Barcelona, Spain; cenjuanes@bellvitgehospital.cat (C.E.); ehidalgoq@bellvitgehospital.cat (E.H.); rramosp@bellvitgehospital.cat (R.R.-P.); sergi.yun@bellvitgehospital.cat (S.Y.); njose@bellvitgehospital.cat (N.J.-B.); 3 Network Research Center on Cardiovascular Diseases (CIBERCV), Instituto de Salud Carlos III, 28029 Madrid, Spain; manuelanguita@secardiologia.es (M.A.); lualmenar@gmail.com (L.A.); marisa.crespo.leiro@sergas.es (M.G.C.-L.); javmu@udc.es (J.M.); 4 Community Heart Failure Unit (UMICO), Cardiology Department, Bellvitge University Hospital, L’Hospitalet de Llobregat, 08097 Barcelona, Spain; 5 Cardiology Department, Reina Sofía University Hospital, IMBIC (Instituto Maimónides de Investigación Biomédica de Córdoba), University of Córdoba, 14004 Cordoba, Spain; 6 Internal Medicine Department, Bellvitge University Hospital, L’Hospitalet de Llobregat, 08907 Barcelona, Spain; fformiga@bellvitgehospital.cat; 7 Heart Failure and Transplant Unit, Cardiology Department, La Fe University Hospital, 46126 Valencia, Spain; 8 Cardiology Department, A Coruña Biomedical Research Institute (INBIC), University Hospital Complex of A Coruña (CHUAC), University of A Coruña (UDC), 15006 A Coruña, Spain; 9 Internal Medicine Department, Ramón y Cajal University Hospital, School of Medicine, University of Alcalá, IRYCIS (Instituto Ramón y Cajal de Investigación Sanitaria), 28034 Madrid, Spain; luis.manzano@uah.es; 10 Instituto de Investigación Biomédica de A Coruña (INIBIC), Universidad de A Coruña, 15006 A Coruña, Spain; 11 Medical Department, Pfizer SLU, 28108 Alcobendas, Spain; jose.chaves@pfizer.com; 12 Department of Clinical Sciences, School of Medicine, University of Barcelona (UB), L’Hospitalet de Llobregat, 08907 Barcelona, Spain

**Keywords:** heart failure, health-related quality of life, obesity, body mass index, body adiposity estimator, patient-centered health outcomes

## Abstract

**Background and Objectives:** Previous studies showed that, paradoxically, obese patients with heart failure (HF) have better clinical outcomes compared to overweight, normal, or underweight patients. Scientific societies emphasize the importance of integrating quality of life (QoL) assessment in cardiovascular care. However, the association between QoL and weight remains understudied. Given the significant correlation between HF survival and QoL, it is essential to assess how obesity impacts patient-reported outcomes in this clinical setting. **Methods:** This cross-sectional multicenter study in 1028 HF patients with reduced ejection fraction (HFrEF) aims to evaluate the association between obesity and QoL, and whether the obesity paradox holds for HF patients regarding QoL. Specific and generic QoL questionnaires were administered alongside clinical parameters like body mass index (BMI) and body adiposity estimator (BAE). **Results:** Obese compared to non-obese reported worse QoL. In the adjusted linear regression models, neither BMI nor obesity were associated with QoL. Generalized additive models confirmed a strong non-parametric association between BMI, subdomain scores from Kansas City Cardiomyopathy Questionnaire (KCCQ) (OSS *p* = 0.004, CSS *p* = 0.006, TSS *p* = 0.02), and summary measurements of EQ-5D (EQ-5D index *p* = 0.003, visual analogue scale (VAS) *p* = 0.01). In contrast, BAE showed a statistically significant linear relation among QoL (OSS *p* ≤ 0.001, CSS *p* ≤ 0.001, TSS *p* ≤ 0.001) and EQ-5D summary measurements (EQ-5D index *p* ≤ 0.001, VAS *p* ≤ 0.001). **Conclusions**: Overall, obese patients have worse QoL; therefore, obesity cannot be considered a protective factor in terms of QoL in established HF.

## 1. Introduction

Obesity is a significant public health problem due to its high incidence and increasing prevalence. Likewise, obesity enhances the risk of cardiovascular disorders, including coronary heart disease and hypertension, which are associated with the expansion of chronic heart failure (HF) [1]. In this regard, HF is a challenging condition due to the negative impact on patients and to public health care systems [2,3].

However, various publications have demonstrated that, paradoxically, obese patients with established chronic HF have better clinical outcomes such as survival in comparison with HF patients who are overweight, normal weight, or underweight. The former phenomenon is known as the “Obesity Paradox” [4,5]. Several pathophysiological hypotheses have been postulated to elucidate this phenomenon, which are essentially related to the neurohormonal activation level in these patients [6,7,8].

Beyond survival, health-related quality of life (HRQoL) is considered a significant clinical outcome with a similar level of relevance to mortality and hospitalization in patients with HF. In recent years, scientific societies and regulatory agencies have stressed the importance of including quality of life (QoL) as a meaningful and necessary clinical endpoint in the evaluation and implementation of therapeutic innovations in the field of cardiovascular medicine [9,10,11,12,13,14].

HRQoL is a patient-reported outcome measure (PROMs) that provides specific information on health status from the patient’s perspective. It integrates multidimensional information that allows us to gauge the limitations imposed by the disease across all the dimensions and domains of daily living that, in turn, shape the patient’s self-perceived health status [1,15,16]. Among the different domains of QoL, physical limitation is the main driver of QoL reported by patients with HF; although, other domains such as social and emotional dimensions have a great influence in the overall perception of health status.

Moreover, QoL has shown a strong association with prognosis and severity in patients with HF. Several studies have shown that worse QoL is associated with clinical outcomes such as mortality [17,18], hospitalization, and medical resource use [18]. In addition, other studies have shown that PROMs strongly correlate with other relevant clinical parameters in HF such as exercise capacity, cardiac and non-cardiac biomarkers, functional parameters [15], psychosocial aspects of the disease [15], and disease experience [19].

However, the associations between QoL and measures of body weight that have shown an association with prognosis, such as body mass index (BMI), adiposity, and obesity, have not been evaluated in depth in patients with HF [5,20,21]. Specifically, it is unclear whether the obesity paradox observed in patients with HF regarding a clinical endpoint such as survival is also applicable to a person-centered clinical endpoint such as QoL. This evaluation is particularly relevant given the strong correlation of survival and other prognostic factors with HRQoL observed in patients with HF [17,18].

Given the gaps of knowledge mentioned above, evaluating the association between BMI, obesity, and QoL, and exploring whether the obesity paradox can be applied in patients with HF regarding PROMs are unmet needs that need to be addressed. We particularly need to know whether BMI and other estimated measures of body composition such as body adiposity correlate with QoL and whether higher values of these parameters and obesity confer a significant benefit in terms of QoL in HF patients.

In this regard, the VIDA-IC study was a multicenter study conducted in Spain that evaluated health-related QoL in more than 1000 patients with systolic HF [15]. The present post hoc analysis of the VIDA study was designed to explore in depth the associations between BMI, estimated adiposity and obesity, and QoL in patients with systolic HF. These analyses will elucidate whether the obesity paradox is also observed for patient-centered outcomes such as QoL.

## 2. Materials and Methods

**Study design.** The methodology of the VIDA-IC study has been previously published [15]. Concisely, the VIDA-IC study was a national, cross-sectional, descriptive observational study in Spain. QoL was assessed in patients with HF using two different instruments. Additional information on important clinical parameters such as weight, height, and consequently body mass index (BMI), were collected prospectively.

**Ethical committee and data availability.** The study was approved by the Ethics and Clinical Research Committee Parc de Salut Mar of the Hospital del Mar Medical Research Institute (IMIM) (PFI-EPL-2011-01), approved on the 10th of July 2011 and was conducted in accordance with the principles of the Declaration of Helsinki. Written informed consent was obtained from all patients before they entered the study.

**Study population and inclusion–exclusion criteria**. HF patients who fulfil the following requirements were included: age ≥ 18 years; diagnosis of chronic HF with reduced left ventricular ejection fraction ≤ 40% (HFrEF) in the last 12 months and stable clinical condition. The criteria for exclusion were patients awaiting heart transplant or valve surgery, those unable to assess or complete QoL questionnaires, extra-cardiac disease and life expectancy less than 1 year, patients who had been hospitalized for non-cardiovascular reasons in the month prior to inclusion, and those hospitalized at the time of inclusion. Patient inclusion was stratified according to recent admission (<1 month) and non-recent (more than 6 months) with a 1:1 ratio for each of the recruiting investigators. Baseline data were collected following informed consent, either from the patient’s medical history or their anamnesis. For the current research, information from 1028 patients out of 1037 of the VIDA-IC study participants was available for analysis.

**Objectives of the study.** The primary objective of the study was to assess the association between BMI and the different dimensions or domains of QoL evaluated by means of the KCCQ and the EQ-5D instruments. The main purpose was to assess whether obese patients reported better health status compared to non-obese patients (obesity paradox in terms of QoL). The secondary exploratory objective was to assess whether BAE was associated with QoL and to define the direction of this association.

**Measurement of patient-centered health outcomes in quality of life.** Every study participant self-administered the Kansas City Cardiomyopathy Questionnaire (KCCQ) [22] and the EuroQol 5D (EQ-5D) [23]. The KCCQ is a reliable tool for evaluating heart failure, composed of twenty-three items grouped into seven dimensions. The outcome for each dimension has a theoretical range from 0 to 100 points, in which a higher score reveals greater health status. Furthermore, the following three summary scores are calculated: the total symptom score (TSS) as a result of the addition of symptom frequency and severity (excluding stability), the clinical summary score (CSS) as a result of the addition of physical and symptom limitation domains, and the overall summary score (OSS) as a result of the addition of clinical summary and quality of life and social limitation domains. The EQ-5D, is a generic tool that consists of a visual analogue scale (VAS) with widely applicable health self-assessment and five domains (mobility, self-care, usual activities, pain/discomfort, and anxiety/depression). The range for VAS scores from 0 (worst state) to 100 (best state). Concerning the rest of the dimensions, the results can be presented as an overall summary index (EQ-5D index) or expressed as the percentage of patients who report any impairment in each dimension. Both scales have been validated in Spain [23]. For the purpose of this analysis, impairment in QoL was defined as QoL scores below the lower tertile.

**Assessment of BMI and body composition.** BMI was calculated as the weight in kilograms divided by the square of the height in meters (kg/m^2^). Obesity was defined as BMI ≥ 30 kg/m^2^. In the study, BMI was divided into five categories based on the non-linear relationship between body mass index and health-related quality of life. Respondents were categorized into: BMI < 20 kg/m^2^, BMI 20.01–25 kg/m^2^, BMI 25.01–30 kg/m^2^, BMI 30.01–35 kg/m^2^, or BMI > 35 kg/m^2^.

Body adiposity estimator (BAE) is a recently described equation assessed by Clínica Universidad de Navarra [24]. BAE is based on BMI, sex, and age for estimating body fat percentage (BF%). The results of BAE were calculated by the subsequent equation: BF% = −44.988 + (0.503 × age) + (10.689 × sex) + (3.172 × BMI) − (0.026 × BMI2) + (0.181 × BMI × sex) − (0.02 × BMI × age) − (0.005 × BMI2 × sex) + (0.00021 × BMI2 × age), where male = 0 and female = 1 for sex, and age in years is developed by multiple regression to predict BF% with a standard error of the estimate of 4.74%. This equation has been shown to be an accurate estimation of BAE in a general population.

**Statistical analysis.** Continuous variables were expressed as mean ± standard deviation. Categorical variables were expressed as n (percentage) and compared by means of the χ^2^ test. Continuous variables were compared applying the Student’s *t*-test or the Mann–Whitney U-test as necessary. Univariable linear regression models, including obesity status (yes vs. no with non-obese as the reference category, or BMI values unit 1 Kg/m^2^) as an independent variable, were conducted to evaluate the clinical and demographic components related to QoL. Based on the univariable linear regression analyses, multivariable models were accomplished with a backward elimination method in order to detect which factors maintained an independent association with QoL, including BMI and BAE as independent variables. Multivariable models were adjusted for variables that showed association with QoL or that have well-known prognostic influence on HF with reduced ejection fraction. These variables were gender, age, number of comorbidities, systolic blood pressure, NYHA functional class, LVEF, serum creatinine, haemoglobin levels, diabetes mellitus, recent HF admission prior to inclusion (<1 month), optimal treatment (number of GMDT—guideline medical treatment drugs), and time since HF diagnosis. Univariable and multivariable generalized additive models (GAM) were used as an alternative to characterize general non-linear regressions. Parametric tests or effects rely on statistical distribution in data whereas non-parametric do not depend on any distribution. Non-parametric (non-linear) *p*-values and parametric (lineal) *p*-values were obtained (if non-parametric *p*-value is less than 0.05, the fitted curve is non-linear and vice versa). GAM models were used to explore the non-parametric associations between the measurements of weight or body composition with HRQoL scores. Finally, adjusted multivariable binary logistic regression analysis was used to explore the association between BMI divided into five categories and QoL impairment, defined as QoL scores below the lower tertile. A *p*-value < 0.05 was considered statistically significant. All statistical analyses were performed using R software (version 4.0.2; R Foundation for Statistical Computing, Vienna, Austria) and SPSS software (version 25.0; IBM, Armonk, NY, USA).

## 3. Results

The VIDA-IC study enrolled 1037 patients, of whom only 9 (0.87%) were excluded from this analysis due to incomplete biometric data. The final cohort of this analysis included 1028 patients.

### 3.1. Baseline Patient Characteristics and Differences Between BMI Categories

Obesity (BMI > 30 kg/m^2^) was observed in 239 (23.2%) patients from the final cohort. BMI was 27.7 ± 4 kg/m^2^ while BAE was 34.1 ± 7% (indicative of body fat). In obese patients, a higher percentage of women was observed (41% vs. 26.7%), as well as a greater proportion of patients with comorbidities (diabetes: 53% vs. 49%, hypercholesterolemia: 79% vs. 65%, sleep apnea: 18 vs. 6.5%, and anemia: 23% vs. 17%). No significant differences were found in age or ejection fraction between the two groups.

Different BMI categories were included in the demographic characteristics of the cohort. We found that 13 (1.3%) patients had a BMI lower than 20 kg/m^2^, 215 (20.9%) patients were between 20.01 and 25 kg/m^2^, and 195 (19%) patients showed a BMI between 30.01 and 35 kg/m^2^. The majority of the sample (561, 54.6% patients) had a BMI from 25.01 to 30 kg/m^2^. The last group, with a BMI higher than 35 kg/m^2^ was composed of 44 (4.3%) patients. Table 1 presents the demographic and clinical characteristics of patients included in the study, both overall and categorized by obesity status.

Obese patients, compared to non-obese, had higher systolic blood pressure and poorer New York Heart Association (NYHA) functional class. Comorbidities such as anemia, diabetes mellitus, or hypercholesterolemia were more common in obese patients.

### 3.2. Analysis of Quality of Life According Obesity Status

**Figure 1:**
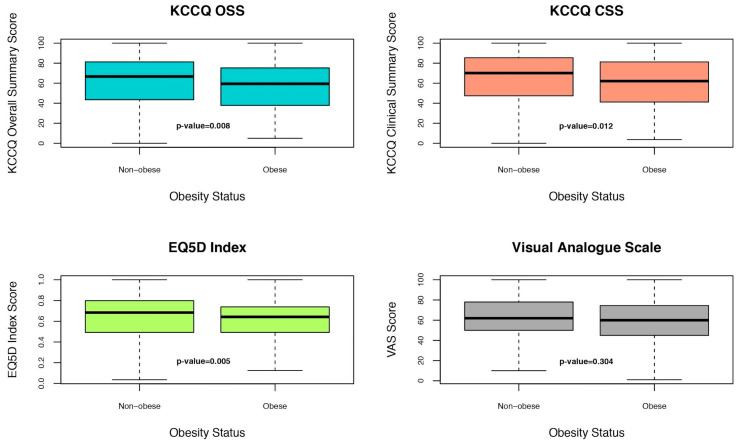
Box plots representing the median and interquartile range of unadjusted QoL scores (KCCQ OSS, ED5D index, EQ5D VAS, and KCCQ clinical summary score) according to obesity status.

Obese patients compared to non-obese reported worse QoL. Figure 1 represents the median and interquartile range of unadjusted QoL scores (KCCQ OSS, KCCQ CSS, EQ-5D index, and EQ-5D VAS) according to obesity status. The same analyses were conducted according to BMI divided in five categories (Supplementary Figure S1).

As shown in Supplementary Table S1, obese patients reported worse QoL depicted by lower scores in the generic (EQ-5D) and disease-specific instruments (KCCQ). Obese patients reported limitations more regularly in all domains of the EQ-5D questionnaire and had lower results in VAS compared to non-obese patients. In this regard, obese patients were more likely to report problems in mobility (66% vs. 56%, *p*-value = 0.005), self-care (66% vs. 56%, *p*-value = 0.005), and pain/discomfort (63% vs. 47%, *p*-value ≤ 0.001). Comparable results were confirmed in the analysis of the KCCQ: obese patients showed lower scores indicating worse HRQoL in the domains reporting on symptom frequency (61.9 ± 26 vs. 68 ± 26, *p*-value = 0.002), burden of symptoms (62.7 ± 27 vs. 68.5 ± 25, *p*-value = 0.002), and social limitation (56.7 ± 29 vs. 63.1 ± 29, *p*-value = 0.004).

Overweight patients (BMI 25.01–30 kg/m^2^), compared to obese, normal-weight, and low-weight patients, obtained better scores in the EQ-5D questionnaire (overall EQ-5D index: 0.66 ± 0.2, *p*-value = 0.02), and in the KCCQ: symptom frequency (68.2 ± 25, *p*-value = 0.019), burden of symptoms (68.9 ± 24, *p*-value = 0.011), and social limitation (63.6 ± 28, *p*-value = 0.021).

### 3.3. Analysis of the Influence of BMI and BAE on QoL

To explore the direct association between obesity and BAE with QoL, an unadjusted linear regression analysis was performed. Obesity status (standardized β coefficient = −0.083, *p*-value = 0.008 for KCCQ OSS; standardized β coefficient = −0.088, *p*-value = 0.005 for EQ-5D index) and higher BMI (standardized β coefficient = −0.056, *p*-value = 0.072 for KCCQ OSS; standardized β coefficient = −0.084, *p*-value = 0.008 for EQ-5D index) were significantly associated with lower QoL scores. Conversely, in the adjusted linear regression models, neither BMI nor obesity were associated with QoL (standardized β coefficient = 0.013, *p*-value = 0.6 for KCCQ OSS; standardized β coefficient = −0.031, *p*-value = 0.3 for EQ-5D index for BMI and standardized β coefficient = −0.016, *p*-value = 0.5 for KCCQ OSS; standardized β coefficient = −0.036, *p*-value = 0.2 for EQ-5D index for obesity).

**Figure 2:**
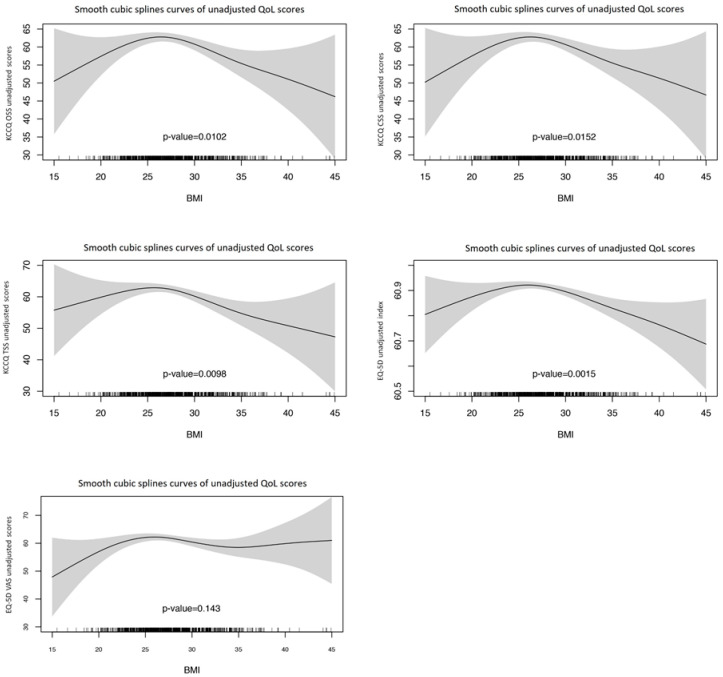
Unadjusted GAM models to explore the parametric and non-parametric associations between BMI and QoL in Overall Summary Score (KCCQ OSS), Clinical Summary Score (KCCQ CSS), Total Symptom Score (KCCQ TSS), EuroQoL 5D index (EQ5Di), and EuroQoL 5D Visual Analogic Scale (EQ5Dvas). *p*-values shown are non-parametric.

**Figure 3:**
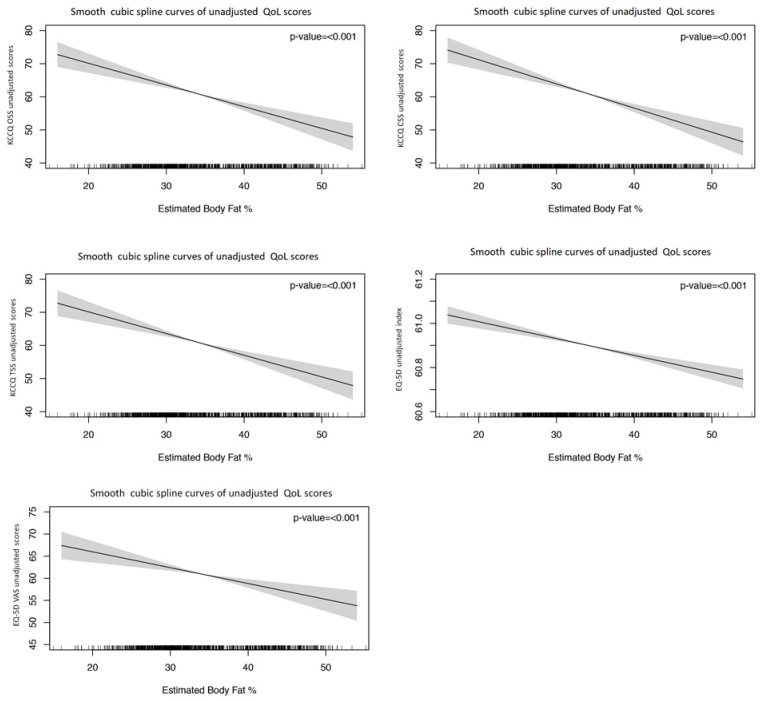
Unadjusted GAM models to explore the parametric and non-parametric associations between BAE and QoL in Overall Summary Score (KCCQ OSS), Clinical Summary Score (CSS), Total Symptom Score (TSS), EuroQoL 5D index (EQ5Di), and EuroQoL. Visual Analogue Scale (EQ5Dvas). *p*-values shown are parametric.

To analyze these findings in more detail, generalized additive models (GAM) were developed to evaluate the non-parametric associations between BMI and BAE with QoL scores. Unadjusted GAM analysis (Figure 2 and Figure 3) showed a strong non-parametric inverse U-shaped association between BMI and QoL. Non-parametric *p*-values were: 0.004 for KCCQ OSS, 0.0065 for KCCQ CSS, 0.0231 for KCCQ TSS, 0.002 for EQ-5D index, and 0.01 for EQ-5D VAS. These results were confirmed in adjusted models (Table 2) evaluating the association between BMI with each of the domains of QoL.

Interestingly, as shown in Figure 3 and Table 2, BAE showed a statistically significant inverse linear association in multivariable GAM with QoL. Parametric *p*-values were: <0.001 for KCCQ OOS, <0.001 for KCCQ CSS, <0.001 for KCCQ TSS, <0.001 for EQ-5D index, and <0.001 for EQ-5D VAS. The smooth cubic splines curves showed an inverse linear association with QoL scores.

Finally, to fully understand the association between BMI and QoL, we additionally conducted an adjusted multivariable binary logistic regression analysis of the association between BMI divided into five categories with impairment of QoL, defined as QoL scores below the lower tertile. As shown in Supplementary Figure S2, patients with BMI between 20.01 and 25 kg/m^2^ showed worse QoL compared to overweight patients (BMI 25.01–30 kg/m^2^), which was the reference category (OSS: OR 2.021, 95% CI (1.267–3.223), *p*-value = 0.003; EQ-index: OR 1.563, 95% CI (0.999–2.444), *p*-value = 0.05; EQ-VAS: OR 1.450, 95% CI (0.948–2.218), *p*-value = 0.08). Similar results were obtained regarding patients with BMI > 35 kg/m2 (OSS: OR 2.672, 95% CI (1.199–5.954), *p*-value = 0.01; EQ-index: OR 2.020, 95% CI (0.919–4.438), *p*-value = 0.08; EQ-VAS: OR 1.963, 95% CI (0.907–4.245), *p*-value = 0.08).

Multivariable models were adjusted for variables that showed association with QoL or that had well-known prognostic influence on HF with reduced ejection fraction. These variables were gender, age, number of comorbidities, systolic blood pressure, NYHA functional class, LVEF, serum creatinine, haemoglobin levels, diabetes mellitus, recent HF admission prior to inclusion (<1 month), optimal treatment (number of GMDT—guideline medical treatment drugs), and time since HF diagnosis.

## 4. Discussion

In this study, we have shown that in HFrEF patients, obese patients have worse QoL compared to non-obese patients. This is the first confirmation that the “obesity paradox” cannot be applied to patients with HF in terms of QoL.

Moreover, we have observed that the association between BMI and QoL is not linear. Indeed, our research showed a non-parametric association between BMI and QoL with an inverse U-shaped association among Spanish adults with HFrEF included in the VIDA-IC study. Moreover, adiposity estimated by BAE further shows a strong inverse linear association with QoL.

Previous studies have reported improved clinical outcomes for obese heart failure patients as compared with their normal weight counterparts [8]. However, to the best of our knowledge, this analysis is the earliest study to investigate the impact of body weight measures on HRQoL [20,21,25,26,27,28].

Only a few studies have evaluated the association between body weight and HRQoL showing mixed results [20,21]. However, these studies were conducted in normal populations, in the primary care setting, without overt cardiovascular disease and, thus, cannot be extrapolated to populations with HF. In these studies, some authors reported a longitudinal association between both parameters (increases in BMI were associated with decreases in physical HRQoL), others suggested an inverse U-shaped association (both underweight and obese respondents had higher risk of scoring lower HRQoL). Our results were in correspondence with the latter findings in general populations.

On the other hand, several studies have explored the association between BMI with functional parameters in HF; however, the results were imprecise. The Obesity Paradox was confirmed for important functional parameters, including LVEF or prognostic biomarkers such as natriuretic peptides or neurohormones [6,7], yet not for exercise tolerance and cardiorespiratory fitness evaluated with peak VO2 [29].

Additional limitations of previous studies such as limited sample sizes [27], no evaluation of HRQoL using validated questionnaires [26,28], no assessment of additional body composition parameters beyond BMI [27,28], no inclusion of patients with established HF in the primary care setting [20], exclusion of underweight patients [28], or selection bias regarding gender [25]. These limitations may have partially contributed to the disparities in the results observed between studies.

These limitations were overcome in our study. In this regard, our study shows for the first time that overweight patients with HFrEF have a better QoL in comparison with low weight and obese patients. It is important to highlight these findings, since these results are based on a large multicenter cohort of patients representative of real-world HF patients.

In this study, we used validated questionnaires to assess HRQoL: KCCQ and EQ-5D. The optimal psychometric properties of these instruments regarding content, validity, and reliability have been widely confirmed in systematic reviews [22,23].

We used BMI as an estimator of body composition. Given the limitations of this parameter, an analysis of the BAE was added as an additional measure of body composition based on BMI, sex, and age for measuring body fat percentage [24]. Our aim was to explore obesity complementing BMI with BAE and correlate the results found in both analyses. In this regard, higher adiposity was linearly associated with lower QoL scores, confirming the absence of obesity paradox for PROMS in the setting of HF. Based on these findings and previous studies [5], it is necessary to reconsider how we evaluate adiposity in our patients, and how imaging techniques can contribute.

The present study contributes to the understanding of the impact of QoL in obese patients with HF-rEF, using real-world data. These findings will help to pave the way to define new components of care and recommendations to be implemented to fulfil the needs of patients according to their perceived QoL and their weight.

Many of the limitations and knowledge gaps observed in previous studies have been addressed in our study. First, we conducted a multicenter study with more than 1000 patients with HFrEF. Second, all patients fulfilling inclusion criteria were evaluated in QoL using generic and disease-specific validated instruments. Third, essential clinical parameters like weight and height were collected [15].

Despite all this, our study leaves a few unanswered questions, including whether these results may be extrapolated to other HF populations such as patients with preserved ejection fraction or whether these findings may be reproduced using more refined body composition estimations. Thus, future investigations are necessary to clarify these unanswered questions.

## 5. Limitations

There are several limitations to our study that require discussion. First, an intrinsic limitation of a cross-sectional evaluation exists. It does not provide information on longitudinal variations of health status or its dynamic interrelations with the clinical features examined over time, and it does not allow drawing causality conclusions. Second, the population included in this research represents a subgroup of patients with HFrEF who are accompanied in hospital outpatient settings.

Third, despite BMI being an important prognostic factor, body composition assessed by a body composition analyzer would have been a more precise measure from the methodological point of view. Fourth, a comprehensive psychosocial and socioeconomic evaluation was not fulfilled in patients recruited in the VIDA-IC study. We do not have any knowledge of whether having incorporated information on health literacy, cognitive function, or social support would have modified the BMI gap in self-perceived heath status observed in our study. These factors might have an impact on impaired HRQoL.

## 6. Conclusions

In this multicenter study involving patients with HFrEF, obese individuals experience lower QoL compared to their non-obese counterparts. This confirms the absence of “The Obesity Paradox” in this context. BMI showed an inverse U-shaped association with QoL for all instruments and in most QoL dimensions. Patients who were overweight had better QoL scores compared to patients with obesity, normal weight, or low weight. Higher levels of adiposity were equally associated with lower levels of QoL. Our findings confirm that obesity cannot be considered a protective factor in terms of HRQoL in established HFrEF.

## Figures and Tables

**Table 1:** Demographic and clinical characteristics of all patients included in this analysis and according to obesity.

	Whole Cohort N = 1028	Non-Obese N = 789	Obese (BMI ≥ 30 kg/m^2^) N = 239	*p*-Value
Demographics				
Age, years	70 ± 11	71 ± 11	69 ± 11	0.066
BMI, kg/m^2^	27.7 ± 4	26.1 ± 2	32.9 ± 3	<0.001
Gender (female) n (%)	309 (30.1)	211 (26.7)	98 (41.0)	<0.001
Systolic blood pressure, mmHg	127 ± 19	126 ± 18	131 ± 20	<0.001
Heart rate, bpm	74 ± 16	74 ± 15	75 ± 16	0.429
NYHA functional class, n (%)				
I–II	546 (54.9)	441 (57.3)	105 (46.7)	<0.001
III–IV	448 (45.1)	328 (42.7)	120 (53.3)	<0.001
Previous hospitalization for HF, n (%)	860 (83.7)	653 (82.8)	207 (86.6)	0.194
Hospitalization for HF < 6 months, n (%)	521 (50.0)	390 (49.4)	131 (54.8)	0.161
Time since HF diagnosis > 1 year, n (%)	695 (67.6)	534 (74.6)	161 (73.2)	0.724
Ischemic etiology, n (%)	521 (50.7)	395 (50.1)	126 (52.7)	0.506
LVEF (%)	34 ± 7	34 ± 6	33 ± 7	0.172
Comorbidities				
Arterial hypertension, n (%)	815 (79.3)	609 (77.2)	206 (86.2)	0.003
Diabetes mellitus, n (%)	450 (43.8)	323 (40.9)	127 (53.1)	0.001
Previous AMI, n (%)	447 (43.5)	339 (43.0)	108 (45.2)	0.552
Hypercholesterolemia, n (%)	704 (68.5)	515 (65.3)	189 (79.1)	<0.001
CKD, n (%)	242 (23.5)	179 (22.7)	63 (26.4)	0.258
Atrial fibrillation, n (%)	449 (43.7)	345 (43.7)	104 (43.5)	1.000
Anemia, n (%)	194 (18.9)	138 (17.5)	56 (23.4)	0.047
COPD or emphysema, n (%)	199 (19.4)	147 (18.6)	52 (21.8)	0.304
TIA/CVA, n (%)	142 (13.8)	104 (13.2)	38 (15.9)	0.286
Pulmonary hypertension, n (%)	204 (19.8)	158 (20.0)	46 (19.2)	0.853
Cancer, no. (%)	81 (7.9)	63 (8.0)	18 (7.5)	0.892
Liver disease, n (%)	43 (4.2)	31 (3.9)	12 (5.0)	0.462
Low weight, n (%)	13 (1.3)	13 (1.6)	0 (0)	0.047
Overweight, n (%)	801 (77.9)	562 (71.2)	239 (100)	<0.001
Sleep apnea, n (%)	94 (9.1)	51 (6.5)	43 (18.0)	<0.001
Anemia, n (%)	194 (18.9)	138 (17.5)	56 (23.4)	0.047
Number of comorbidities, points	4.7 ± 2	4.5 ± 2	5.4 ± 2	<0.001
Treatment				
ACEi or ARBs	922 (89.7)	702 (89.0)	220 (92.1)	0.184
Beta blockers	787 (76.6)	600 (76.0)	187 (78.2)	0.542
MRA	684 (66.5)	520 (65.9)	164 (68.6)	0.481
Ivabradine	91 (8.9)	72 (9.1)	19 (7.9)	0.697
Digital	221 (21.5)	171 (21.7)	50 (20.9)	0.858
Diuretics	917 (89.2)	703 (89.1)	214 (89.5)	0.906
Statins	779 (75.8)	586 (74.3)	193 (80.8)	0.047
Antiplatelet agents	618 (60.1)	467 (59.2)	151 (63.2)	0.291
Anticoagulants	410 (39.9)	309 (39.2)	101 (42.3)	0.407
Laboratory				
Hemoglobin, g/dL	12.9 ± 1.7	12.9 ± 1.7	12.8 ± 1.7	0.483
Creatinine mg/dL	1.31 ± 0.8	1.29 ± 0.76	1.37 ± 0.90	0.184
Creatinine clearance mL/min/1.73 m^2^	61.2 ± 27	61.1 ± 26	61.3 ± 31	0.954
Creatinine clearance <60, n (%)	259 (45)	191 (44)	68 (49)	0.328
NT-proBNP, pg/mL, (median, IQR)	1135 [503–2392]	410 [260–1700]	618 [395.25–2148]	0.809
BNP, (pg/mL) (median, IQR)	218.5 [124–430.50]	198 [107–596]	112 [93.5–181]	0.003

BMI: body mass index. NYHA: New York heart association. HF: heart failure. LVEF: left ventricular ejection fraction. AMI: acute myocardial Infarction. CKD: chronic kidney disease, defined as estimated glomerular filtration (eGFR) date < 60 mL/min/1.73 m^2^. COPD: chronic obstructive pulmonary disease. TIA: transient ischemic attack. CVD: cerebrovascular disease. ACEi: angiotensin-converting enzyme inhibitors. ARBs: angiotensin receptor blockers. MRA: mineral-corticoid receptor antagonists. IQR: interquartile range.

**Table 2:** Adjusted GAM models to explore the parametric and non-parametric associations between BMI and BAE with QoL.

Dependent Variables	BMI		BAE	
KCCQ	Parametric *p*-Value	Non-Parametric *p*-Value	Parametric *p*-Value	Non-Parametric *p*-Value
Subdomain Score				
Physical limitation	0.3382	0.004297	<0.001	0.6476
Stability of symptoms	0.3632	0.04502	0.443	0.321
Symptom frequency	0.02026	0.02452	<0.001	0.3157
Burden of symptoms	0.01003	0.03059	<0.001	0.6089
Self-efficacy	0.02703	0.2681	0.001102	0.01074
Quality of life	0.2715	0.01515	0.0001383	0.3082
Social limitation	0.05831	0.003436	<0.001	0.4025
KCCQ, summary measures				
Overall summary score	0.07085	0.004032	<0.001	0.5897
Clinical summary score	0.07325	0.006469	<0.001	0.602
Summary symptom score	0.01206	0.02308	<0.001	0.464
EQ-5D, summary measurements				
Overall EQ-5D index	0.008009	0.002694	<0.001	0.3393
Visual analogue scale	0.5857	0.01461	<0.001	0.1583

KCCQ: Kansas City Cardiomyopathy Questionnaire. EQ-5D: EuroQol 5 dimensions.

## Data Availability

The original data presented in the study are included in the article, further inquiries can be directed to the corresponding authors.

## Supplementary Materials

The following supporting information can be downloaded at: https://www.mdpi.com/article/10.3390/jcm13247558/s1.
