# Supplementary material for: No Obesity Paradox for Health-Related Quality of Life in Patients with Heart Failure and Reduced Ejection Fraction: Insights from the VIDA Multicenter Study
Source: J Clin Med. 2024 Dec 12;13(24):7558. doi: 10.3390/jcm13247558 (PMC11728257; doi:10.3390/jcm13247558)

Supplementary Table S1. Distribution of summary scores and dimensions or domains of the HF-specific (KCCQ) and generic (EQ-5D) QoL instruments used in the study according to the presence or absence of obesity.

KCCQ	n	Global	Non-Obese (BMI<30 kg/m²)	Obese(BMI≥30 kg/m²	p-value
**Subdomain Score**					
Physical limitation	1023	61.1±28	61.9±28	58.4±28	0.086
Stability of Symptoms	1022	59.5±23	59.8±23	58.8±24	0.567
Symptom Frequency	1027	66.4±26	67.7±26	61.9±26	0.002
Burden of Symptoms	1027	67.2±26	68.5±25	62.7±27	0.002
Self-efficacy	1026	69.1±22	69.6±22	67.2±24	0.147
Quality of life	1026	54.4±24	55.2±24	51.7±24	0.055
Social Limitation	1018	61.6±29	63.1±29	56.7±29	0.004
**KCCQ, summary measures**					
Overall Summary Score	1014	60.9±24	62±24	57.2±24	0.008
Clinical Summary Score	1023	63.9±25	65±25	60.4±25	0.012
Total Symptom Score	1027	66.8±25	68.1±25	62.3±26	0.002
**EQ-5D, % patients reporting problems**					
Mobility, n (%)	1001	581(58.0)	424(56)	157(66)	0.005
Self-care, n (%)	1001	581(58.0)	424(56)	157(66)	0.005
Usual activities, n (%)	1000	615(61.5)	457(60)	158(66)	0.080
Pain/Discomfort, n (%)	999	506(50.7)	356(47)	150(63)	<0.001
Anxiety/Depression, n (%)	999	490(49.0)	361(47)	129(54)	0.075
**EQ-5D, summary measurements**					
Overall EQ-5D Index	993	0.646±0.3	0.659±0.3	0.6055±0.2	0.005
Visual Analogue Scale	1013	60.7±19	61.1±20	59.6±20	0.304

Supplementary Figure S1. Box plots representing the median and interquartile range of unadjusted QoL scores (KCCQ OSS, KCCQ CSS, ED5D index and EQ5D VAS) according to BMI divided in 5 categories.

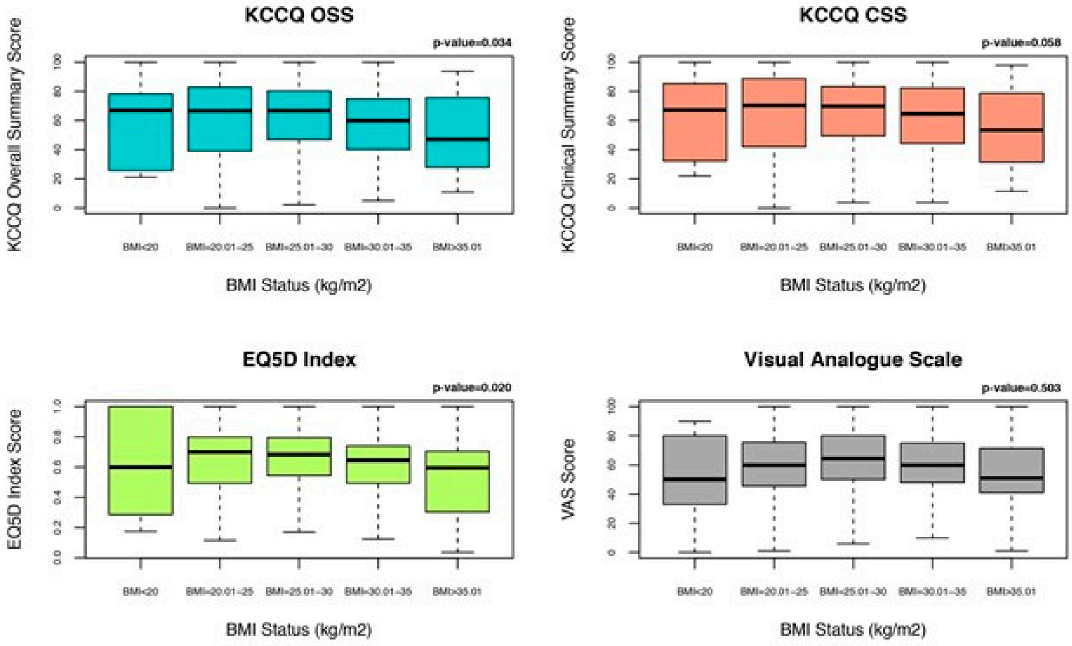

Supplementary Figure S2. Multivariate binary logistic regression models for the evaluation of the association between BMI divided in 5 categories and the presence of impairment in QoL (defined as QoL scores below the lower tertile. BMI categories: BMI 1 (<20 kg/m2), BMI 2 (20.01-25 kg/m2), BMI 3 (reference category, 25.01-30 kg/m2), BMI 4 (30.01-35 kg/m2), BMI 5 (>35 kg/m2).

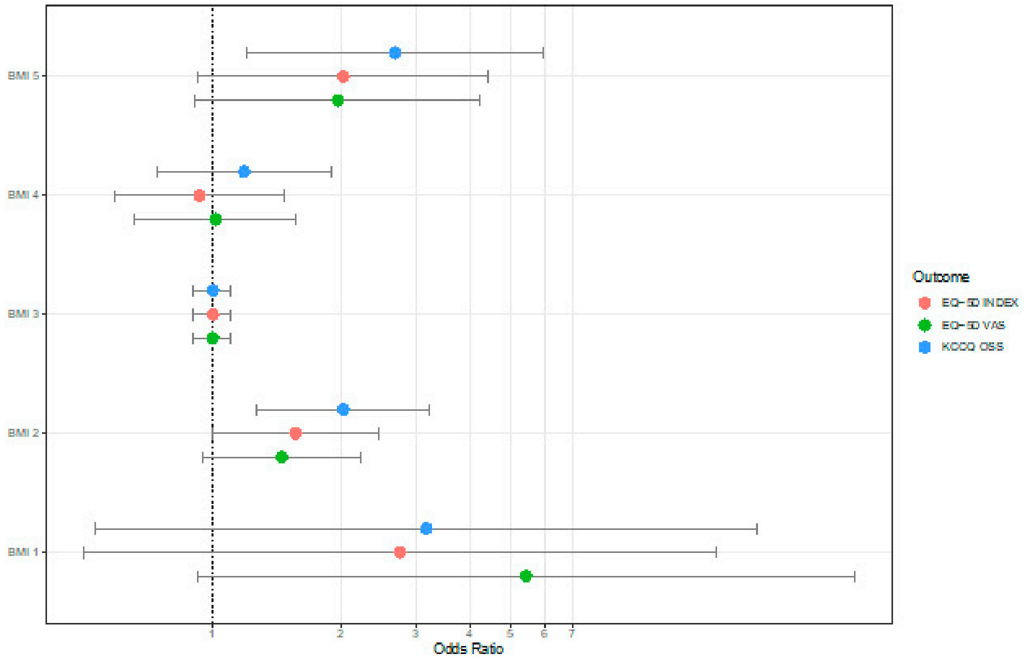

Supplement: Supplementary file 1 [file jcm-13-07558-s001.zip › jcm-3310815-supplementary.pdf]
